# An autophagy-related molecule reticulon 3 functions as a novel prognostic biomarker in hepatocellular carcinoma

**DOI:** 10.1016/j.gendis.2025.101929

**Published:** 2025-11-08

**Authors:** Zhu Xie, Wei Wang, Peng Yu, Mingdong Zhang, Zixin Hu, Hongyan Wang

**Affiliations:** a Department of Ophthalmology, Ninth People’s Hospital, Shanghai Jiaotong University School of Medicine, Shanghai 200240, China; b Department of General Dentistry, Shanghai Ninth People’s Hospital, Shanghai Jiao Tong University School of Medicine, College of Stomatology, Shanghai Jiao Tong University, National Center for Stomatology, National Clinical Research Center for Oral Diseases, Shanghai Key Laboratory of Stomatology, Shanghai 200011, China; c Department of Cardiology, Zhongshan Hospital, Fudan University, Shanghai Institute of Cardiovascular Diseases, Shanghai 200032, China; d Department of Thoracic Surgery, Jiading Branch of Shanghai General Hospital, Shanghai Jiaotong University School of Medicine, Shanghai 201803, China; e Artificial Intelligence Innovation and Incubation Institute, Fudan University, Shanghai 201203, China; f Shanghai Academy of Artificial Intelligence for Science, Shanghai 201111, China; g Obstetrics and Gynecology Hospital, State Key Laboratory of Genetics and Development of Complex Phenotypes, Shanghai Key Laboratory of Metabolic Remodeling and Health, Institute of Metabolism and Integrative Biology, Fudan University, Shanghai 200438, China; h Shenzhen Maternal and Child Health Hospital, Shenzhen, Guangdong 518000, China

Hepatocellular carcinoma (HCC) is a highly lethal malignant tumor, and its unique pathology leads to limited therapeutic benefits.^1^ Autophagy plays a pivotal role in cellular homeostasis, facilitating macromolecule and energy recycling and conferring protection against cellular stress. Autophagy exerts a dual role in cancer initiation and progression. In the initiation phase of tumorigenesis, it can clear pathogenic mutant proteins and prevent the accumulation of harmful substances that damage DNA, thus inhibiting tumor formation. In the cancer progression phase, autophagy may supply nutrients for synthetic metabolism in cancer cells, fostering tumor development. Furthermore, activating autophagy can enhance the sensitivity of cancer cells to chemotherapy and amplify the anti-tumor effects of chemotherapeutic agents.^2^^,^^3^ Therefore, profiling the function and prognostic value of autophagy-related genes (ARGs) is critical to characterize new biomarkers and prognostic risk models for HCC management. Here, we conducted a detailed investigation into the single-cell expression profiling, function, and genetic alterations of ARGs using transcriptomic data from patients within the TCGA-LIHC cohort. DNA methylation analysis uncovered novel methylation sites significantly correlated with patient survival outcomes. We then developed a prognostic model based on ARGs expression through univariate COX regression analysis. Kaplan–Meier survival analysis and receiver operating characteristic (ROC) curve revealed that the risk model could exactly predict the prognosis of HCC patients. Employing machine learning approaches, including LASSO, Random Forest, and Support Vector Machine algorithms, we identified reticulon 3 (RTN3) as a key protein with prognostic significance. Both RTN3 expression and the risk score were found to be independent indicators of immune cell infiltration within the tumor microenvironment. Furthermore, molecular docking and kinetic simulation experiments suggested ivermectin as a potential therapeutic agent targeting RTN3. Collectively, our findings reveal novel biomarkers, a robust prognostic model, and a candidate drug, offering new insights into HCC management.

We studied the molecular features and functions of ARGs in HCC. We found significant changes in 12 out of 60 ARGs we analyzed.^4^ Single-cell expression profiles and functional analysis showed that these genes were closely related to autophagy and were mostly expressed in hepatocytes, which interact strongly with immune cells (Fig. 1A–D; Fig. S1A–S1D, S2). We also analyzed single-nucleotide polymorphisms and DNA methylation levels of ARGs in HCC. Results showed that 51% of HCC patients had mutations in at least one of these genes, with ATG2B having the highest mutation rate (Fig. S1E). Methylation levels of 22 ARGs were significantly higher, while 20 genes had lower methylation (Table S1). Hypomethylation of 27 genes was linked to poorer patient outcomes, while hypermethylation of 20 genes was associated with better survival (Table S2).

**Figure 1:**
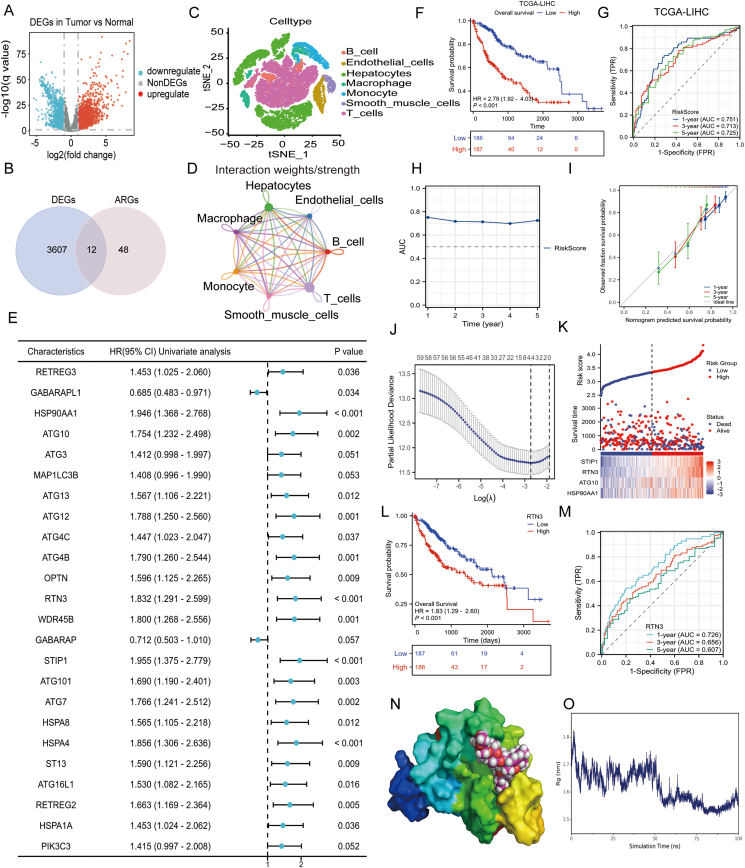
Integrative systematic analysis of the functional and prognostic value of autophagy-related genes (ARGs) in hepatocellular carcinoma (HCC). **(A)** Volcano plot illustrating differentially expressed genes (DEGs). The *x*-axis represents the log2 fold change in gene expression, while the *y*-axis denotes the –log10 transformed *P*-values, indicating statistical significance. Genes that are up-regulated in HCC are marked in red, down-regulated genes in blue, and non-differentially expressed genes in grey. **(B)** Venn diagram comparing ARGs with DEGs. The exclusive DEGs are represented in blue (*n* = 3607), exclusive ARGs in red (*n* = 48), and the intersection, indicating genes that are both DEGs and ARGs, is shown in the overlapping area (*n* = 12). **(C)** Downscaled visualization plots depicting single-cell cluster clustering using t-Distributed Stochastic Neighbor Embedding (tSNE). **(D)** Network diagram representing cell–cell interactions. Nodes correspond to different cell types, and connecting lines, along with their thickness, signify the degree of interaction between cells. **(E)** Forest plots summarizing the outcomes of univariate Cox regression analyses. **(F, G)** Kaplan–Meier survival curves and receiver operating characteristic (ROC) curves for patients in the TCGA-LIHC dataset. **(H)** Time-dependent area under the curve (AUC) changes in risk scores. **(I)** Calibration curves for the multifactorial prognostic analysis. The *x*-axis represents the nomogram-predicted survival probability, while the *y*-axis corresponds to the actual observed survival probability. The ideal line indicates perfect concordance between predicted and observed probabilities. Color-coded dots and error bars correspond to the predicted versus actual survival probabilities at 1, 3, and 5 years, respectively. **(J)** The relationship between partial likelihood deviance (PLD) and the logarithm of the regularization parameter λ (Log(λ)), with the optimal λ range demarcated by two dotted lines. **(K)** Heatmap depicting risk score distribution, patient survival status, and key gene expression. The upper dot plot uses blue for the low-risk group and red for the high-risk group, with dot positions corresponding to risk scores. The middle dot plot indicates patient survival status (blue for deceased, red for alive). The lower heatmap shows key gene expression levels, with color intensity representing expression levels. **(L)** Kaplan–Meier survival curve based on RTN3 gene expression. The blue line represents the low-expression group, and the red line represents the high-expression group. **(M)** ROC curves for RTN3 gene expression in predicting patient survival at 1, 3, and 5 years. **(N)** Visualization of the protein-ligand complex using a surface model for the protein and a ball-and-stick model for the ligand. **(O)** Display of the radius of gyration (Rg) as a function of simulation time. The Rg metric quantifies the compactness of the molecular structure, offering a measure of its overall shape and size fluctuations throughout the simulation.

We developed a risk-prognostic model for HCC based on ARGs expression. Using univariate COX regression, we identified 24 risk genes and stratified HCC patients in the TCGA-LIHC dataset into high- and low-risk groups according to their risk scores (Fig. 1E; Fig. S3E). Kaplan–Meier survival analysis and ROC curves revealed that high-risk patients had significantly shorter survival times and that the model exhibited high predictive accuracy (Fig. 1F–H; Fig. S3F). These findings were validated in the ICGC-LIRI-JP-HCC and GSE14520 datasets (Fig. S3A–S3D). Decision curve analysis demonstrated that combining independent risk factors with patient tumor status effectively assessed clinical benefits (Fig. S3G, S3I, S3J). Calibration curves and nomogram analyses further confirmed that ARGs expression accurately predicted patient survival, even when integrated with other clinical factors (Fig. 1I; Fig. S3H).

To identify key autophagy-related prognostic genes in HCC, we employed three machine learning models: LASSO, Random Forest, and Support Vector Machine. By integrating the simulation results from three different models, we highlighted RTN3 as a critical factor in HCC prognosis (Fig. 1J and K; Fig. S3K–S3M). Kaplan–Meier survival and ROC curve analyses revealed that high RTN3 expression correlates with shorter patient survival and accurately predicts prognosis (Fig. 1L, M; Fig. S3N). To explore the clinical implications of RTN3 expression and risk factors, we assessed immune infiltration levels in HCC patients. High-risk groups exhibited elevated infiltration of dendritic cells, T helper 2 cells, and regulatory T cells (Fig. S4A–S4D). Conversely, tumor-suppressive immune cells, such as naïve B cells and naïve natural killer cells, were less abundant in the high RTN3 expression group (Fig. S4E–S4I). We further examined the polarization of macrophages and neutrophils in the tumor microenvironment of patients with high RTN3 expression, using TGFB1 and IFNG expression as indicators. Elevated TGFB1 expression in this group suggests a tumor-promoting phenotype of macrophages and neutrophils, which may contribute to the poorer survival outcomes observed in patients with high RTN3 expression (Fig. S4J, S4K).

To identify potential small-molecule compounds targeting RTN3 for HCC treatment, we employed molecular docking and modeling techniques. Initially, we retrieved 3D structures of chemical molecules known to interact with RTN3 from the PubChem database. Subsequent molecular docking analyses evaluated the binding affinity of these compounds to RTN3, leading us to select ivermectin as a candidate for further kinetic simulations.^5^ These simulations revealed that the ivermectin–RTN3 complex was structurally stable and intact, with dynamic internal residue changes indicating an active region (Fig. 1O; Fig. S5A–S5C). Hydrogen bonding and force analyses showed that the complex formed a stable number of hydrogen bonds and exhibited strong van der Waals interactions between key amino acids (Fig. 1N; Fig. S5D–S5J). Collectively, these results suggest a robust interaction between ivermectin and RTN3, providing a molecular basis for ivermectin as a targeted therapy for HCC.

In summary, our study provides valuable insights into the role of ARGs in HCC by identifying novel biomarkers, developing a robust prognostic model, and pinpointing a potential therapeutic target. The discovery of new methylation sites and their correlation with patient survival outcomes highlights the importance of epigenetic modifications in HCC prognosis. The prognostic model based on ARGs expression demonstrates high accuracy in predicting patient outcomes, potentially aiding in clinical decision-making and patient stratification. The identification of RTN3 as a key prognostic protein underscores its significance in tumor progression. Furthermore, the suggestion of ivermectin as a potential therapeutic agent targeting RTN3 offers a promising direction for future drug development. Overall, our findings not only enhance the understanding of the molecular mechanisms underlying HCC but also provide new tools and strategies for improving the diagnosis, prognosis, and treatment of HCC. However, our study has certain limitations. For instance, in the GSE14520 dataset, the relatively small sample size and incomplete clinical data led to suboptimal accuracy in the survival prediction of hepatocellular carcinoma patients using our risk prognostic model. This underscores the need for further validation of our findings in larger cohorts. Additionally, our investigation of RTN3’s function is limited to computational analyses, and further *in vitro* and *in vivo* experimental validation is required to provide stronger support for our findings.

## CRediT authorship contribution statement

**Zhu Xie:** Writing – review & editing, Writing – original draft, Visualization, Validation, Resources, Project administration, Methodology, Investigation, Formal analysis, Data curation, Conceptualization. **Wei Wang:** Writing – review & editing, Writing – original draft, Resources, Methodology, Investigation. **Peng Yu:** Writing – review & editing, Writing – original draft, Resources, Methodology, Investigation. **Mingdong Zhang:** Writing – review & editing, Writing – original draft, Methodology, Investigation. **Zixin Hu:** Writing – review & editing, Writing – original draft, Project administration, Methodology, Conceptualization. **Hongyan Wang:** Writing – review & editing, Writing – original draft, Project administration, Funding acquisition, Conceptualization.

## Funding

This work was jointly supported by grants from the Basic Science Center Program (China) (No. 32488101 to H.W.), Shenzhen Medical Research fund (China) (No. B2402004 to H.W.), Sanming Project of Medicine in Shenzhen, Guangdong, China (No. SZSM202311005 to H.W.), Shanghai Municipal Science and Technology Major Project (China) (No. FCK-92724413-2024-025 to H.W.), and the China Postdoctoral Science Foundation (No. 2025M772792 to Z.X.).

## Conflict of interests

The authors declared no competing interests.
