# Supplementary material for: An autophagy-related molecule reticulon 3 functions as a novel prognostic biomarker in hepatocellular carcinoma
Source: Genes Dis. 2025 Nov 8;13(5):101929. doi: 10.1016/j.gendis.2025.101929 (PMC13126270; doi:10.1016/j.gendis.2025.101929)
Supplement: Multimedia component 1 [file mmc1.docx]

**Supplementary information**

**Materials and Methods**

**Data Extraction and differential analysis**

A total of 60 autophagy-related genes (ARGs) was identified from the previous report. Gene expression profiles and corresponding clinical data from hepatocellular carcinoma (HCC) patients, comprising 50 normal and 371 tumor samples, were extracted from the TCGA-LIHC cohort. Expression profiles and survival data from the ICGC-LIRI-JP cohort and the GSE14520 dataset served as validation sets for the prognostic model. The limma R package was utilized to identify differentially expressed genes (DEGs), applying a threshold of absolute log2-fold change (FC) > 0.5 and an adjusted P-value < 0.05.

Single-cell RNA sequencing (scRNA-seq) data for four patients were sourced from GSE146115. We processed the data using the Seurat package, with the number of highly variable genes set to 3,000. Subsequently, the data were scaled and underwent principal component analysis (PCA). Cluster analysis was conducted using the 'FindNeighbors' and 'FindCluster' functions within Seurat. Annotation of distinct cell types was facilitated by the R package 'SingleR'. Dimensionality reduction was achieved using the t-distributed Stochastic Neighbor Embedding (tSNE) method.

**Functional Enrichment Analysis**

Biological functional enrichment analysis was conducted utilizing Gene Ontology (GO) and Gene Set Enrichment Analysis (GSEA), while Kyoto Encyclopedia of Genes and Genomes (KEGG) analysis was performed using the ClusterProfiler package. Enriched pathways were visualized with the 'ggplot2' R package. Pathways were considered significant with P < 0.05.

**Machine learning model construction**

Support Vector Machine-Recursive Feature Elimination (SVM-RFE) and random forest algorithms were deployed to identify diagnostic markers for hepatocellular carcinoma (HCC). The 'caret' package was used to implement SVM-RFE with recursive feature selection and K-fold cross-validation, with k set to 10. The 'randomForest' package facilitated the construction of a random forest model, which was trained on 1000 distinct combinations of features. Classification outcomes were derived from the consensus of the individual trees within the forest. The accuracy of the classification model was evaluated using the out-of-bag estimation error rate.

**Establishment and verification of the prognostic model**

Univariate Cox proportional hazards regression analysis was employed to identify candidate prognostic genes, visualized with the 'survival' and 'forestplot' R packages. The risk score, a predictive factor, was calculated as the sum of the products of the coefficients and the expression levels of the associated genes:

Risk score=$\sum_{i=1}^{n} \mathrm{Coef}i\times Xi$, where Coef*_i_* is the regression coefficient of the prognostic gene and *X_i_* is its expression level. Subsequently, the least absolute shrinkage and selection operator (LASSO) was used to further filter the prognostic genes for model establishment, multivariate Cox proportional hazards regression analysis was applied to discern critical clinical phenotypes. Patients in both the TCGA-LIHC cohort and validation datasets were stratified into high- and low-risk groups based on the median risk score. The predictive efficacy of the model was depicted using Kaplan-Meier survival curves generated with the 'survminer' R package. Endpoints at 1, 3, and 5 years were established, and the model's performance was appraised using time-dependent ROC curves, implemented with the 'timeROC' R package. A nomogram was constructed to predict overall survival based on the risk score and clinicopathological characteristics, including age, gender, and stage, utilizing the 'rms' R package.

**Immune cell infiltration analyses**

The CIBERSORT, QUANTISEQ, MCPCOUNTER, XCELL, and ssGSEA algorithms were utilized to compare immune cell infiltration between high-risk and low-risk groups, stratified by the autophagy-related genes (ARGs) signature. The results are depicted through heatmap and violin plot illustrations.

**Molecular docking and Molecular dynamics simulations**

The molecular structures of compound Ivermectin were retrieved from the PubChem database. Crystal structures of the target proteins were obtained from the Research Collaboratory for Structural Bioinformatics Protein Data Bank (RCSB PDB). Before proceeding with molecular docking, proteins were prepared by eliminating any existing ligands and water molecules using PyMOL. Subsequently, the proteins underwent further processing in AutoDock Tools, where desolvation and hydrogenation were performed to refine the structures for docking studies. Visualization of the two-dimensional (2D) and three-dimensional (3D) docking results was carried out using Discovery Studio 2024 and PyMOL.

Molecular dynamics (MD) simulations were conducted using the AMBER20 software suite with the FF14SB force field. The simulations were initialized with the FF14SB force field and temperature was regulated using the Berendsen coupling integration algorithm to maintain a constant temperature throughout the simulation. Analysis of the simulation outcomes was performed using the PTRAJ module within the AMBER20 software suite, while Xmgrace was used for the calculation and visualization of four key attributes. The stability and dynamics of the docked complexes were assessed over a 60-nanosecond simulation period, with parameters including Root Mean Square Deviation (RMSD), Root Mean Square Fluctuation (RMSF), and Radius of Gyration (RoG) being evaluated to predict the behavior of the complexes.

**Statistical analysis**

Statistical analyses were conducted using GraphPad Prism and R software. Data presented are the average of at least three independent experiments, each performed in triplicate, and are reported as mean ± standard deviation (SD). For comparisons between two groups, Student's t-test was applied. One-way analysis of variance (ANOVA) was utilized for multiple group comparisons. P-value < 0.05 was considered to indicate statistical significance.

**Figure and Legends**


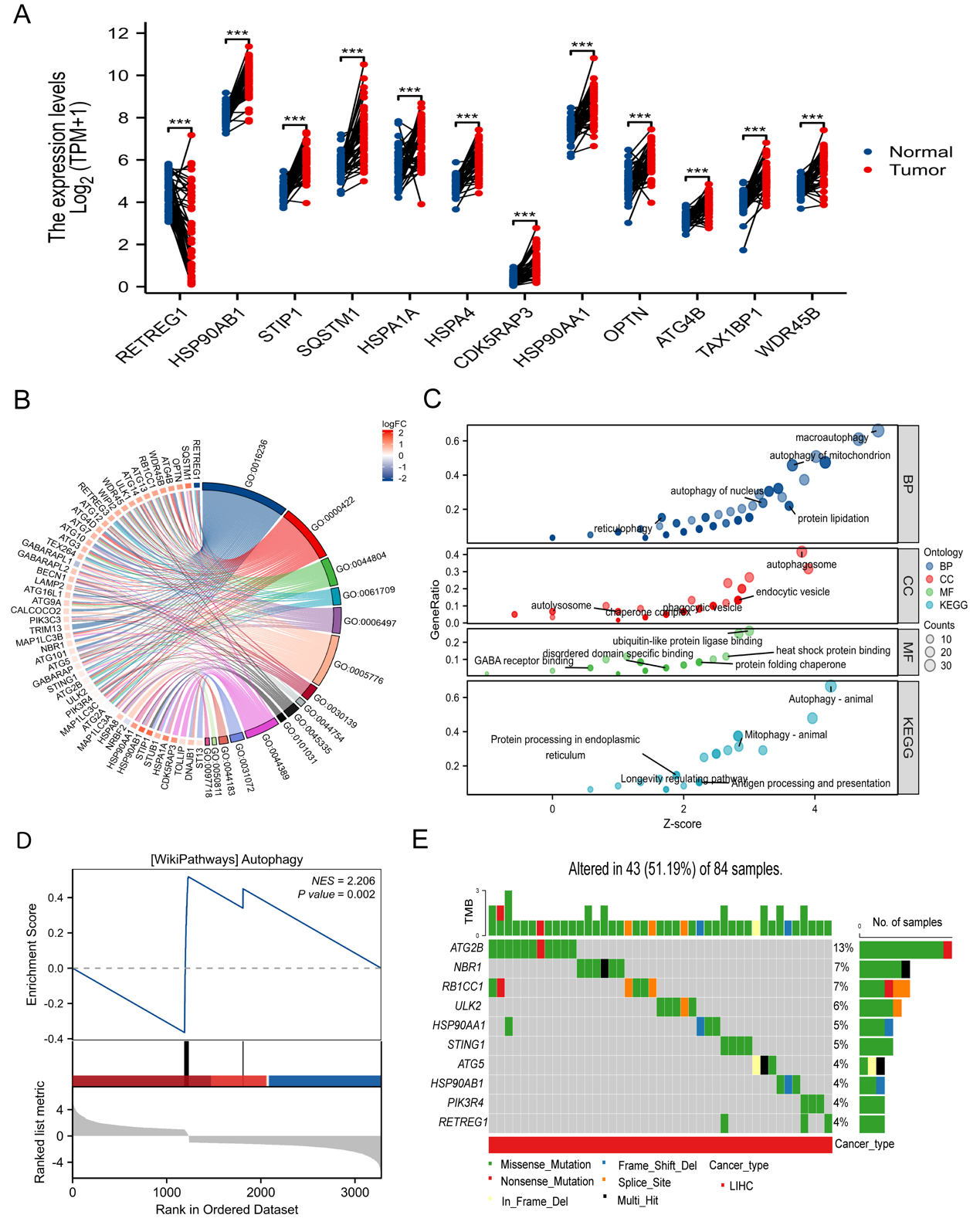


**Figure S1.** Differential gene expression, functional characterization and mutation analysis of autophagy-related genes in HCC. (**A)** Violin plot showcasing the expression levels of DEGs in paired normal and tumor tissues. Data distribution is indicated by black dots and lines. **(B)** String diagram illustrating the functional enrichment of autophagy-related genes. This visualization maps the distribution and interconnections of these genes across various functions and cellular compartments. The outer ring denotes gene identifiers, while the inner ring categorizes them according to Gene Ontology (GO) classes. The connecting lines and color gradients, which correspond to log2 fold change (logFC) values, signify the strength of association between genes and their respective GO annotations. (**C)** Bubble diagram depicting the functional enrichment of autophagy-related genes. The diagram presents enrichment in terms of bubble positioning (Z-score) and size (gene count) across four GO categories: biological process (BP), cellular component (CC), molecular function (MF), and KEGG pathways. **(D)** Gene Set Enrichment Analysis (GSEA) plot highlighting the enrichment scores of autophagy-related gene sets within the dataset. The plot captures the variation in enrichment scores, providing insights into the collective behavior of gene sets related to autophagy. (**E)** Waterfall plot showcasing the mutational landscape of autophagy-related genes in hepatocellular carcinoma. The tumor mutation burden (TMB) is indicated at the top of the plot. Different colors at the bottom represent distinct mutation types, while the bars on the right side quantify the variant allele frequency of each gene within the samples. *** p< 0.001.


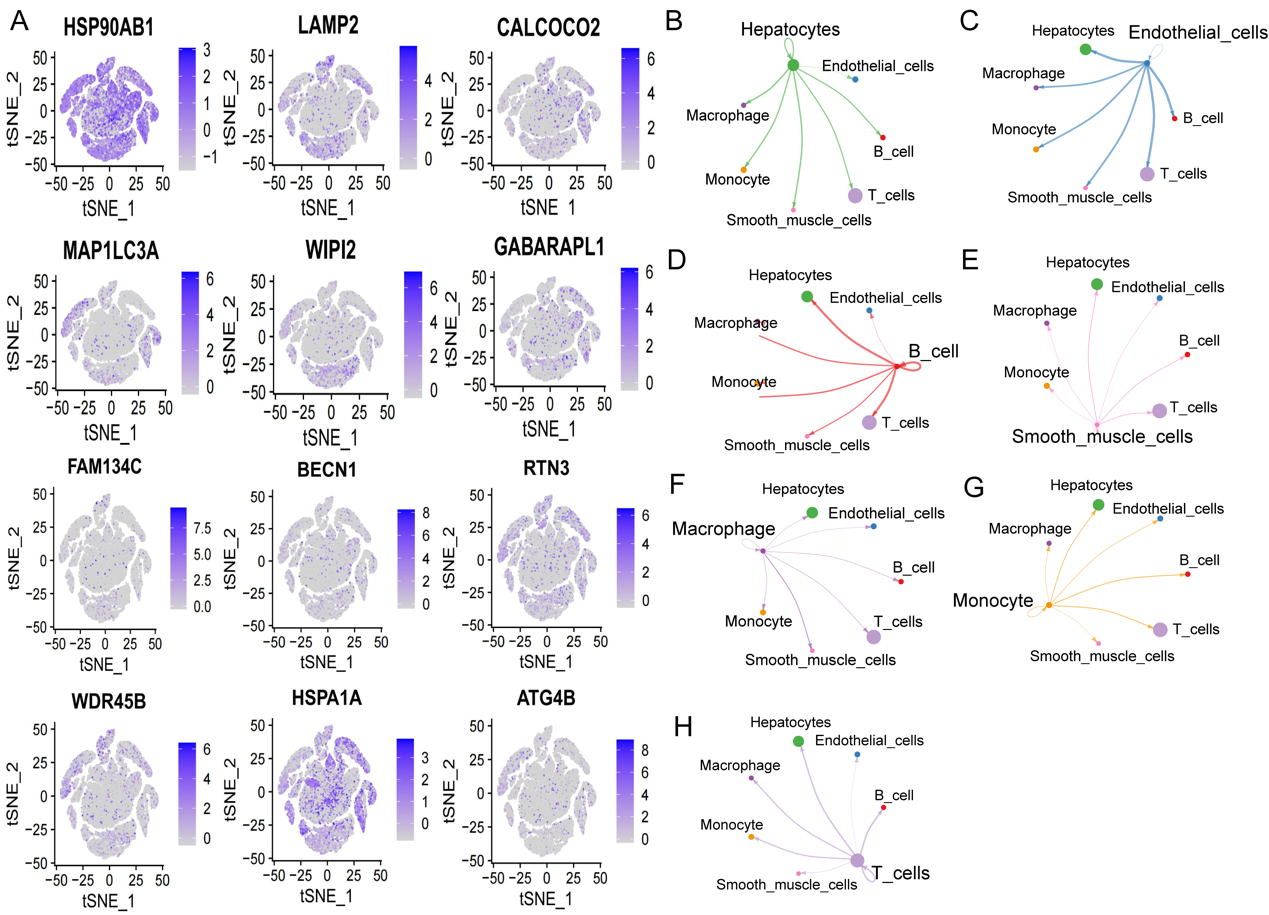


**Figure S2.** Single-cell expression profiles and cellular interactions of ARGs. **(A)** Gene expression visualization graph based on tSNE and ISNE (Iterative Stochastic Neighbor Embedding) dimensionality reduction. Each small graph within the panel illustrates the expression level of a specific gene across different cell types. The color intensity, with darker shades indicating higher expression, allows for the observation of expression differences among various cell types. **(B-H)** Network diagrams detailing specific cellular interactions.


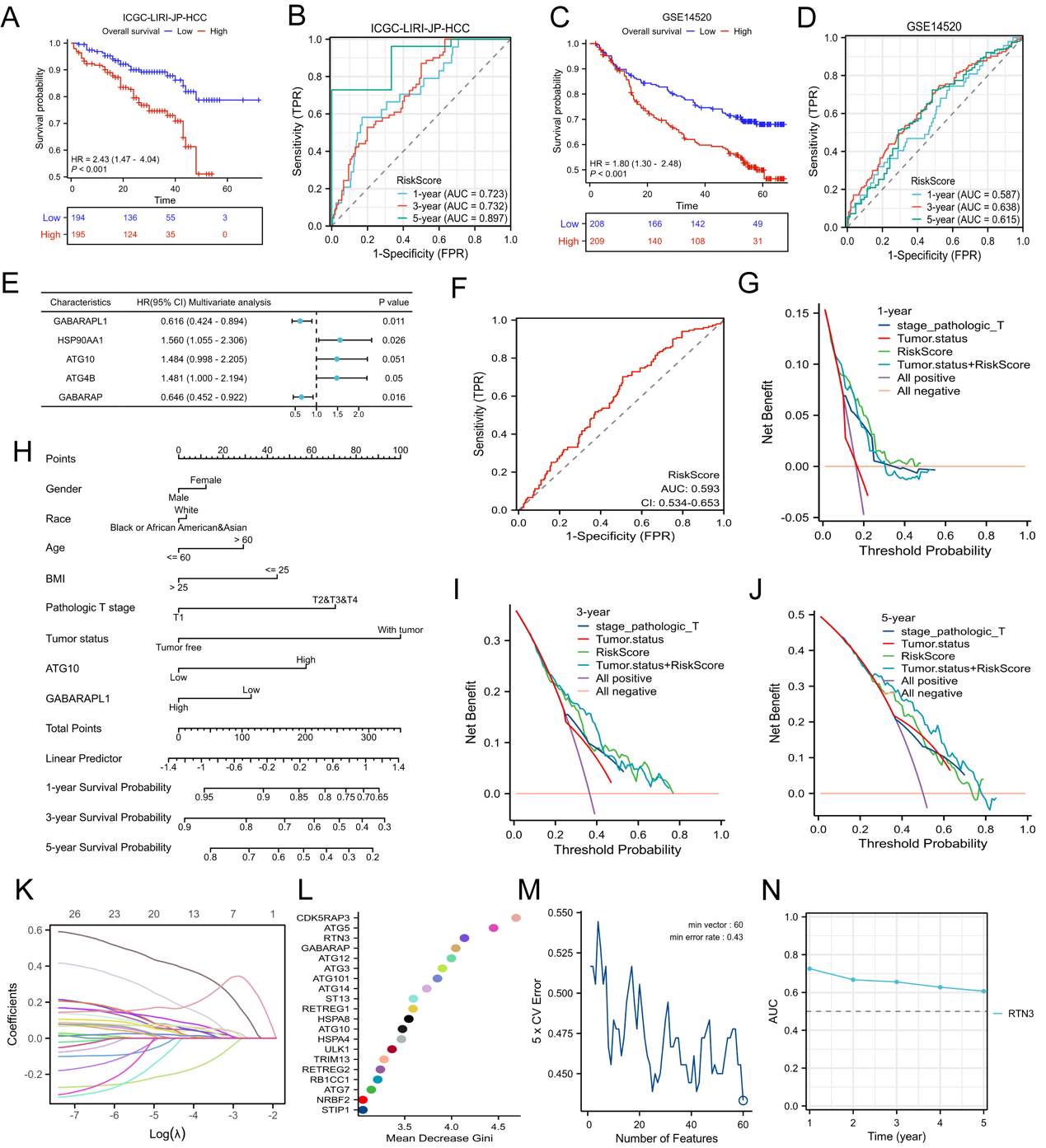


**Figure S3.** Assessment of ARGs Expression as a Prognostic Indicator and identification of Key Prognostic genes in HCC. (**A, B)** Kaplan-Meier survival curves and ROC curves for patients in the ICGC-LIRI-JP-HCC dataset. **(C, D)** Kaplan-Meier survival curves and ROC curves for patients in the GSE14520 dataset. Additional validation is provided by assessing the prognostic performance in yet another independent cohort. **(E)** Forest plots summarizing the outcomes of multivariate Cox regression analyses. **(F)** ROC curves changes in risk scores. **(G)** Nomogram depicting the impact of multifactorial predictors on patient survival probability. This plot quantifies the relative contribution of each prognostic factor to the overall survival probability, providing insights into their clinical relevance. **(H-J)** Decision curve analysis (DCA) plots evaluating the clinical factors of pathological T-stage, tumor status, risk score, the combination of tumor status and risk score, and the binary extremes of "All positive" and "All negative". The x-axis represents the Threshold Probability, and the y-axis represents the Net Benefit. These curves allow for a comparative assessment of the net benefit conferred by each predictor across various threshold probabilities, thereby determining their clinical utility. (**K)** displays the coefficient profiles of various genes across different λ values, highlighting their variable importance. (**L)** Assessment of gene importance using Mean Decrease Gini. The y-axis lists gene names, while the x-axis represents Mean Decrease Gini values. Genes are color-coded, with larger values indicating greater importance in the model. (**M)** Five-fold cross-validation (5×CV) error plot to determine the optimal number of features. The x-axis shows the number of features, and the y-axis represents the 5×CV error rate. The plot is annotated with the minimum vector (min vector) and minimum error rate (min error rate), guiding the selection of the most informative features. (**N)** Changes in AUC values of RTN3 gene expression at different time points, assessing the stability of its predictive ability across various time spans.


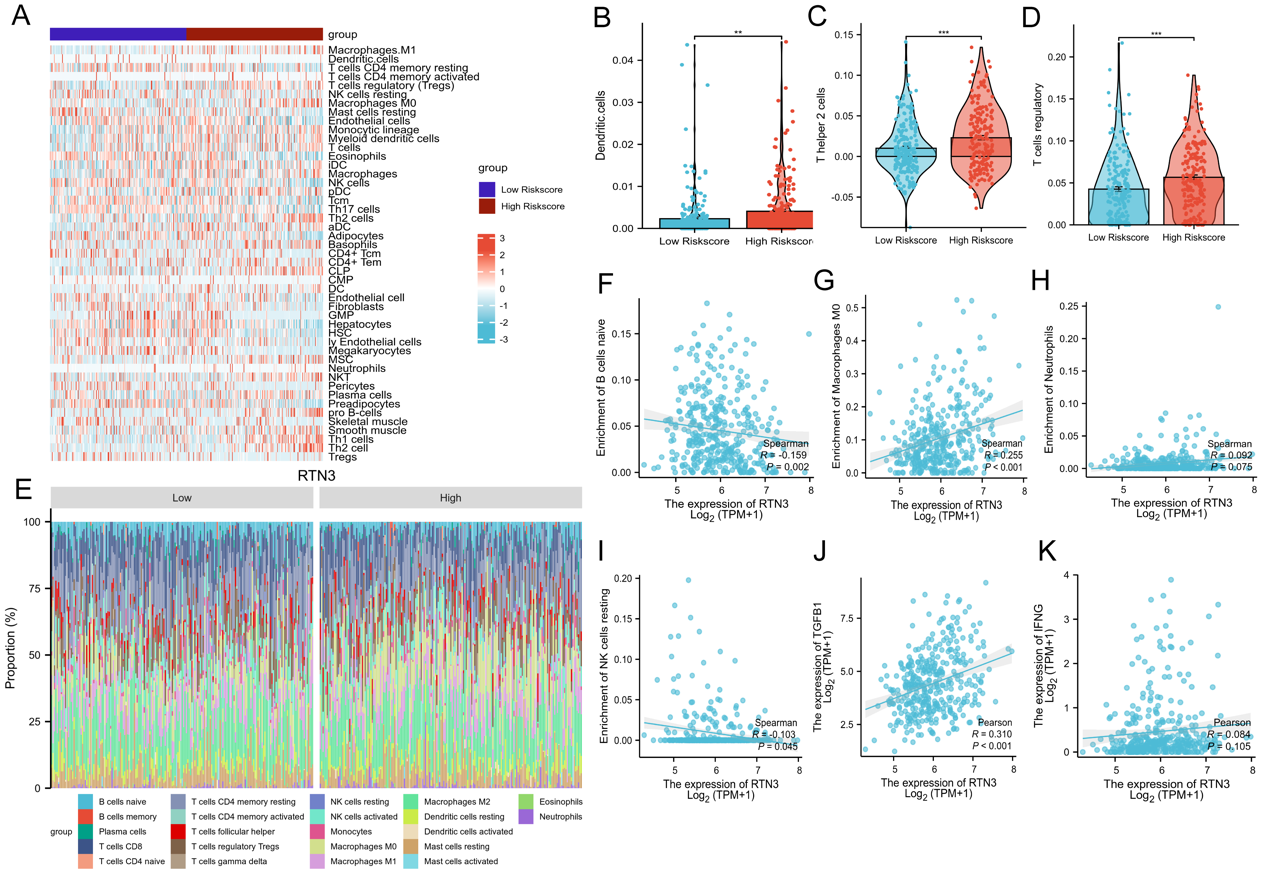


**Figure S4.** Immune Cell Infiltration and Correlations with RTN3 Expression in Hepatocellular Carcinoma. (**A)** Heatmap illustrating the levels of immune cell infiltration in high- and low-risk groups. The color intensity represents the percentage of immune cells. **(B-D)** Violin plots depicting the distribution of different immune cell percentages in high- and low-risk groups. The black dots and lines within the plots denote the distributional characteristics of the data. **p< 0.01, ***p< 0.001. (**E)** Stacked bar graph of immune cells, stratified by RTN3 gene expression levels. The colors representing different immune cell types to highlight the correlation between RTN3 gene expression and immune cell composition. (**F-K)** Scatter plots analyzing the correlations between RTN3 gene expression and specific immune cell types or gene expressions. Spearman or Pearson correlation coefficients and p-values are provided to indicate the strength and significance of these correlations.


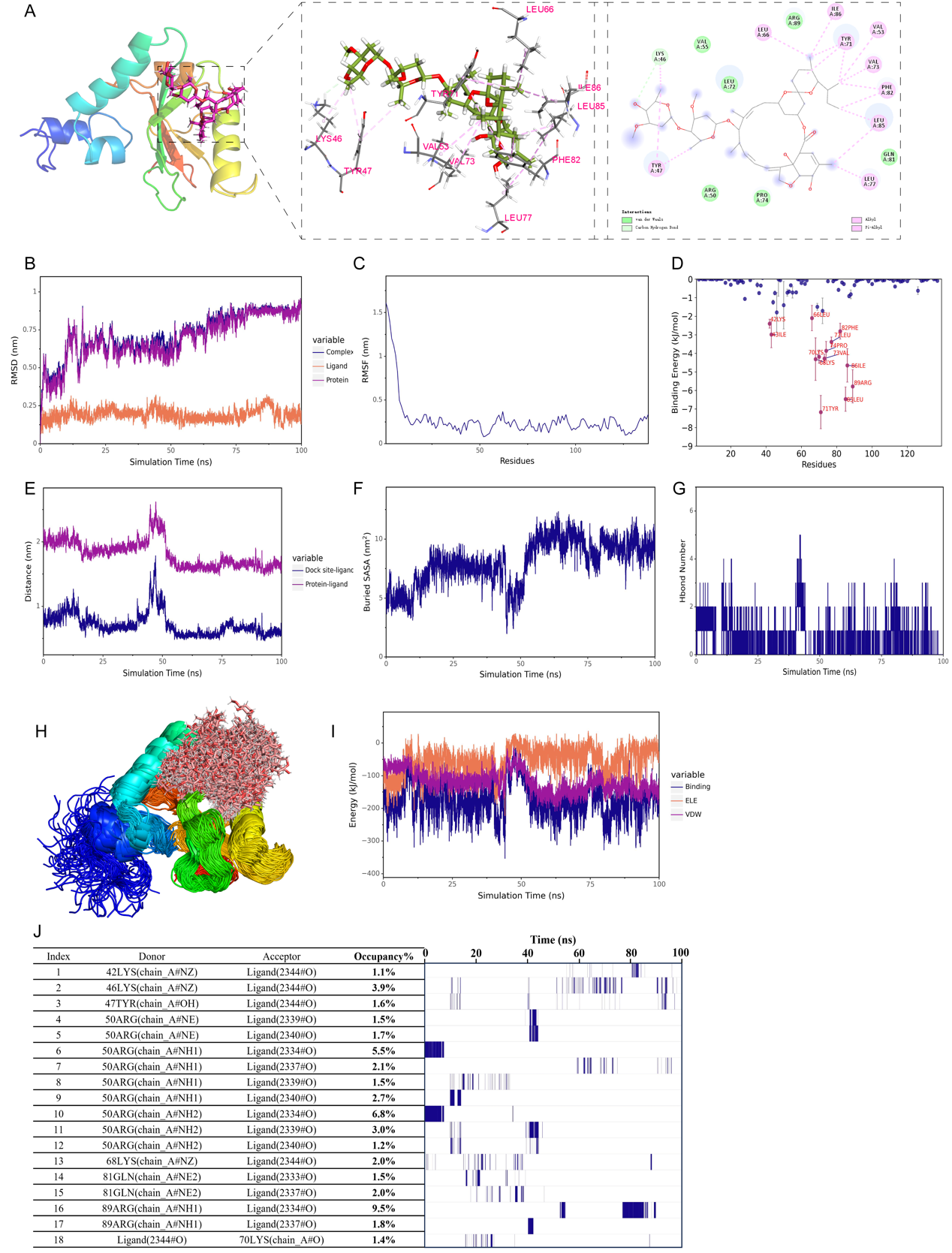


**Figure S5.** Structural and Dynamic Analysis of the Protein-Ligand Complex. **(A)** Schematic representation of the protein-ligand complex architecture. The left panel presents the overall three-dimensional structure of the protein, color-coded to delineate distinct structural domains. The central panel zooms in on the ligand-binding site, depicting the atomic details in a stick model with key amino acid residues annotated. The right panel illustrates the ligand-amino acid interactions in a two-dimensional format, highlighting hydrogen bonds with pink dashed lines and hydrophobic interactions with green circles. (**B)** Plot of Root Mean Square Deviation (RMSD) against simulation time, detailing the RMSD trajectories for the complex, ligand, and protein individually. (**C)** Presentation of Root Mean Square Fluctuation (RMSF) values for individual amino acid residues. The x-axis lists amino acid residue numbers, while the y-axis shows the corresponding RMSF values. (**D)** Bar graph depicting the contribution of each amino acid residue to the binding energy. The x-axis corresponds to amino acid residue numbers, and the y-axis represents the magnitude of binding energy. Notably, red dots denote key residues that exert a substantial influence on the overall binding energy, underscoring their critical role in the protein-ligand interaction. **(E)** Displays the temporal evolution of distances between the ligand and the docking site (dark blue curve) as well as between the protein and the ligand (purple curve) over a 100 ns simulation period. (**F)** Illustrates the solvent accessible surface area (SASA) of the ligand as it changes over time during the simulation. (**G)** Depicts the variation in the number of hydrogen bonds throughout the simulation period. (**H)** Presents the three-dimensional structure of the protein-ligand complex. The protein's structural domains are color-coded, and the ligand is highlighted in red, offering a clear visualization of the spatial arrangement and interaction interface between the two molecules. (**I)** Shows the change in binding energy (blue), electrostatic energy (ELE, orange), and van der Waals energy (VDW, purple) over the simulation time. (**J)** Enumerates the different hydrogen bonds by their donor, acceptor, and occupancy percentage. The accompanying time series graph on the right documents the presence of each hydrogen bond over the course of the simulation, highlighting their temporal stability and contribution to the overall interaction.
